# PERCEIVED NEIGHBORHOOD SOCIAL DISORDER, FRIENDS’ SUPPORT, AND DEPRESSIVE SYMPTOMATOLOGY OF NON-MARRIED OLDER WOMEN

**DOI:** 10.1093/geroni/igz038.1142

**Published:** 2019-11-08

**Authors:** Seungjong Cho, Aloen Townsend

**Affiliations:** Case Western Reserve University, Cleveland, Ohio, United States

## Abstract

This study examined whether perceived neighborhood social disorder predicted depressive symptoms among non-married older women (N = 823) drawn from the 2016 Health and Retirement Study. It also tested the stress buffering effect of friends support. A negative binomial regression model showed that higher perceived neighborhood social disorder was associated with higher depressive symptoms. Presence of good friends in the same neighborhood and number of close friends were protective factors, but no stress buffering effect of friends support was identified. This study highlights the adverse effect of negative perceptions of the neighborhood social environment on non-married older women’s depressive symptoms.

